# AMP‐dependent kinase stimulates the expression of αKlotho

**DOI:** 10.1002/2211-5463.13872

**Published:** 2024-08-01

**Authors:** Julia Vogt, Lisa Wolf, Ludwig E. Hoelzle, Martina Feger, Michael Föller

**Affiliations:** ^1^ Department of Physiology University of Hohenheim Stuttgart Germany; ^2^ Institute of Animal Science, University of Hohenheim Stuttgart Germany

**Keywords:** CKD, FGF23, longevity, metformin, phenformin

## Abstract

Renal αKlotho along with fibroblast growth factor 23 regulates phosphate and vitamin D metabolism. Its cleavage yields soluble Klotho controlling intracellular processes. αKlotho has anti‐inflammatory and antioxidant effects and is nephro‐ and cardioprotective. AMP‐dependent kinase (AMPK) is a nephro‐ and cardioprotective energy sensor. Given that both αKlotho and AMPK have beneficial effects in similar organs, we studied whether AMPK regulates αKlotho gene expression in Madin–Darby canine kidney, normal rat kidney 52E, and human kidney 2 cells. Using quantitative real‐time PCR and western blotting, we measured αKlotho expression upon pharmacological manipulation or siRNA‐mediated knockdown of AMPKα. AMPK activator 5‐aminoimidazole‐4‐carboxamide ribonucleoside (AICAR) enhanced αKlotho expression, an effect reduced in the presence of AMPK inhibitor compound C or siRNA targeting AMPK catalytic α‐subunits (α1 and α2). Similarly, AMPK activators metformin and phenformin upregulated αKlotho transcripts. Taken together, our results suggest that AMPK is a powerful inducer of αKlotho and could thereby contribute to the development of future therapeutic interventions.

## Graphical abstract

We show that AMP‐dependent kinase (AMPK) is a powerful inducer of αKlotho in three cell lines. 5‐aminoimidazole‐4‐carboxamide ribonucleoside (AICAR) is a rather specific AMPK activator while metformin and phenformin are unspecific AMPK activators with several off‐target effects. Images provided by Servier Medical Art (https://creativecommons.org/licenses/by/4.0/) and adapted under a Creative Commons Attribution 4.0 Unported License.

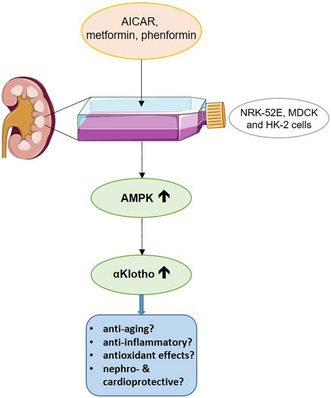

**Abbreviations**
ACC: acetyl‐CoA carboxylase
AKI: acute kidney injury
AICAR: 5‐aminoimidazole‐4‐carboxamide ribonucleoside
AMP: adenosine monophosphate
AMPK: AMP‐dependent kinase
CKD: chronic kidney disease
FGF23: fibroblast growth factor 23
HK‐2: human kidney 2
IGF1: insulin‐like growth factor 1
MDCK: Madin–Darby canine kidney
NRK‐52E: normal rat kidney 52E
PI3K: phosphoinositide 3‐kinase
sKl: soluble Klotho
Tbp: TATA box‐binding protein
Wnt: wingless and Int‐1

Renal transmembrane protein αKlotho has several important functions [1]. On the one hand, it serves as a co‐receptor for phosphaturic hormone fibroblast growth factor 23 (FGF23), which is mainly produced in bone [2, 3, 4]. On the other hand, enzymatic cleavage of transmembrane αKlotho results in an extracellular form, called soluble Klotho (sKL), which exerts endocrine and paracrine effects in several tissues and organs [5]. FGF23 exerts further effects in other organs including heart [6] and is correlated with outcomes in kidney [7] and cardiovascular disease [8].

The joint action of FGF23 and αKlotho in the kidney is pivotal for phosphate and vitamin D metabolism [9, 10, 11, 12]. Lack of either FGF23 or αKlotho results in massive phosphate and 1,25(OH)_2_D_3_ (active vitamin D) excess in mice, causing a phenotype of rapid aging with a plethora of aging‐associated diseases that are reminiscent of human aging and affect almost all tissues and organs [13, 14, 15]. Conversely, overexpression of αKlotho has powerful antiaging effects, expanding the life span by about 30% in mice [16].

αKlotho has not only strong antiaging properties, but has also been demonstrated to be highly beneficial in several acute and chronic disorders: It is nephroprotective (e.g., by preventing renal fibrosis in chronic kidney disease (CKD)) [17], cardioprotective (e.g., by inhibiting cardiac remodeling or cardiac fibrosis) [18], or has multiple anticancer effects as a tumor suppressor (e.g., by inhibiting Wnt or insulin‐like growth factor 1 (IGF1)‐dependent phosphoinositide 3‐kinase (PI3K) signaling) [19, 20, 21]. Further putatively health‐promoting effects of αKlotho may include the reduction in oxidative stress or anti‐inflammatory effects to name a few [22, 23, 24].

AMP‐dependent kinase (AMPK) is basically expressed in all cell types and consists of three subunits, α, β, γ [25]. Physiologically, it is activated by increase in cellular AMP concentration, indicating lack of ATP and hence energy deficiency [26]. In rough summary, AMPK reduces cellular processes consuming energy and induces pathways providing energy [27]. Higher AMPK activity is associated with some remarkable health benefits. These may include the protection of the heart during ischemia, the reduction of microvascular disease in diabetes, or nephroprotection in insulin resistance [28, 29, 30]. These beneficial effects are largely attributed to improvements in cell metabolism or stimulation of autophagy [30, 31]. Interestingly, metformin, a drug discussed to have antiaging properties and to expand life span [32], is an activator of AMPK [33].

Given the fact that both αKlotho and AMPK have beneficial effects in similar organs, we wondered whether AMPK is a regulator of αKlotho. Using NRK‐52E, MDCK, and HK‐2 cells, we tested the effect of AMPK activity on αKlotho gene expression.

## Materials and methods

### Cell culture and treatments

Madin–Darby canine kidney (MDCK, NBL‐2) cells (CVCL_0422; Cell Lines Services, Eppelheim, Germany) were cultured in Dulbecco's modified Eagle medium F12 (DMEM/F‐12; Gibco, Life Technologies, Darmstadt, Germany) containing 3.1 g/L glucose, 5% fetal bovine serum (FBS) (Gibco), 100 U/mL penicillin, and 100 μg/mL streptomycin (Gibco), as well as 2 mm glutamine (Gibco). Normal rat kidney 52E (NRK‐52E) cells (ECACC 87012902; European Collection of Authenticated Cell Cultures, Salisbury, UK) were cultured in Dulbecco's modified Eagle medium (DMEM) with 4.5 g/L glucose and supplemented with 5% newborn calf serum, 100 U/mL penicillin, and 100 μg/mL streptomycin (all reagents from Gibco). For experiments, cells were plated at a density of 1.5 × 10^5^ cells per well (MDCK) or 2 × 10^5^ cells per well (NRK‐52E) in 6‐well tissue culture plates (Greiner Bio‐One, Frickenhausen, Germany) and kept at 5% CO_2_ and 37 °C. After 24 h, cells were treated with or without AMPK activator AICAR (1 mm; Selleckchem, München, Germany) for further 24 h (MDCK) or 48 h (NRK‐52E) in the presence of serum and in the absence or presence of 20 μm (MDCK) or 1 μm (NRK‐52E) AMPK inhibitor compound C (Tocris; Bio‐Techne, Wiesbaden, Germany). MDCK cells were treated with the indicated concentrations of biguanides metformin hydrochloride or phenformin (both from PeproTech, Hamburg, Germany) for 24 h. Human kidney 2 (HK‐2) cells (CRL‐2190; American Type Cell Collection (ATCC), Manassas, VA, USA) were cultured in DMEM with 4.5 g/L glucose containing 10% FBS, 100 U/mL penicillin, and 100 μg/mL streptomycin. Cells were seeded at a density of 1.2 × 10^5^ cells per well into 6‐well plates and grown for 24 h. Next, HK‐2 cells were treated with 1 mm AICAR (48 h) or 3 mm metformin hydrochloride (24 h).

### Silencing

For silencing, 1.2 × 10^5^ MDCK cells per well were seeded into 6‐well culture plates and grown for 24 h in complete medium. Next, cells were transfected with custom‐designed AMPKα_1_ (12.5 nm) and AMPKα_2_ (12.5 nm) small interfering RNA (siRNA) (Invitrogen; Thermo Fisher Scientific, Darmstadt, Germany), or 25 nm nontargeting control siRNA (siNeg) (#4390843; Invitrogen) using 5 μL lipofectamine RNAiMAX (Invitrogen) transfection reagent in antibiotic‐free complete medium. For silencing of AMPKα in NRK‐52E cells, 1.5 × 10^5^ cells per well were grown in complete medium for 24 h. Cells were transfected with custom‐designed AMPKα_1_ (25 nm) and AMPKα_2_ (25 nm) siRNA (Invitrogen), or 50 nm nontargeting control siRNA (siNeg) (#4390843; Invitrogen) with 5 μL DharmaFECT 1 transfection reagent (Horizon Discovery, Cambridge, UK) in antibiotic‐free complete medium. Twenty‐four hours after transfection, 1 mm AICAR was added and the cells were incubated for further 24 h (MDCK) or 48 h (NRK‐52E). Silencing efficiency was verified by quantitative real‐time PCR (qRT‐PCR).

### Quantitative real‐time PCR (qRT‐PCR)

Cells were washed with ice‐cold PBS and total RNA was isolated with TriFast reagent (Peqlab, Erlangen, Germany) according to the manufacturer's manual. Isolates from MDCK cells were additionally treated with DNase, and RNA was extracted using NucleoSpin RNA Mini kit (Macherey‐Nagel, Düren, Germany). Total RNA (1.2 μg; 60 ng/μL) was used for cDNA synthesis with random primers and the GoScript Reverse Transcription System (Promega, Mannheim, Germany; 25°C for 5 min, 42°C for 1 h, and 70°C for 15 min). qRT‐PCR was performed on the CFX Connect Real Time System (Bio‐Rad Laboratories, Feldkirchen, Germany) using GoTaq qPCR Master Mix (Promega). qRT‐PCR conditions were as follows: 95°C for 5 min, followed by 40 cycles of 95°C for 10 s, annealing at primer‐specific temperature for 30 s, and 72°C for 30 s. The final volume of the qRT‐PCR master mix was 20 μL containing 2 μL cDNA, 0.25 μm (canine *KL*, *PRKAA1*, *PRKAA2*, rat *Kl* and human *KL*) or 0.5 μm (rat *Tbp*, *Prkaa1*, *Prkaa2*, *Nfe2l2*, canine *TBP*, and human *TBP*) of a specific primer pair for the target gene, and nuclease‐free water.

Calculated mRNA expression levels were normalized to TATA box‐binding protein (TBP) transcripts. Relative quantification of target gene expression was performed by the 2^−∆CT^ method. The primer sequences are presented in Table 1.

**Table 1:** Primer sequences for quantitative real‐time PCR (qRT‐PCR) analyses.

Gene	Species	Forward primer (5′ → 3′)	Reverse primer (5′ → 3′)	Reference sequence	Annealing temperature
*TBP*	*dog*	CCTATTACCCCTGCCACACC	GCTCCCGTACACACCATCTT	XM_038684469.1	60 °C
*TBP*	*human*	TGCACAGGAGCCAAGAGTGAA	CACATCACAGCTCCCCACCA	NM_001172085.2	59 °C
*Tbp*	*rat*	ACTCCTGCCACACCAGCC	GGTCAAGTTTACAGCCAAGATTCA	NM_001004198.1	57 °C
*KL*	*dog*	AAATGAAGCTCTGAAAGCC	AATGATAGAGGCCAAACTTC	XM_038434663.1	56 °C
*KL*	*human*	TGGAAACCTTAAAAGCCATCAAGC	CCACGCCTGATGCTGTAACC	NM_004795.4	59 °C
*Kl*	*rat*	CAACTACATTCAAGTGGACC	CAGTAAGGTTTTCTCTTCTTGG	XM_038458582.1	54 °C
*PRKAA1*	*dog*	AAGATAGCTGATTTTGGTCT	CTCCATATATCTACCTCTGG	XM_038535247.1	56 °C
*Prkaa1*	*rat*	CTCAACCGGCAGAAGATTCG	TGGAACAGACGTCGACTCTC	NM_019142.3	58 °C
*PRKAA2*	*dog*	ATCTGTAAACATGGACGGGTTGA	ATGTGAGCATCCAACAGCAC	XM_038520518.1	56 °C
*Prkaa2*	*rat*	GGAGGGTTGAAGAGGTGGAA	TCCGGTGCTGCATAATTTGG	XM_039110823.1	58 °C
*Nfe2l2*	*rat*	CCATTTGTAGATGACCATGAG	GTATTAAGACACTGTAACTCGG	NM_001399173.1	57 °C

### Western blotting

NRK‐52E, HK‐2 and MDCK cells were seeded into T25 cell culture flasks or 6‐well culture plates (Greiner Bio‐One) under standard cell culture conditions. After 24 h or after silencing, cells were treated with 1 mm AICAR for 6 h and 48 h (NRK‐52E), 24 h (MDCK) or 38 h (HK‐2), 3 mm metformin hydrochloride for 24 h (HK‐2), or with vehicle only. Cells were lyzed using ice‐cold RIPA buffer (Cell Signaling Technology, Danvers, MA, USA) supplemented with protease and phosphatase inhibitor cocktail and EDTA (Halt; Thermo Fisher Scientific), and 30 μg of total protein was used for 8% or 10% SDS/PAGE and subsequent standard western blotting. Antibodies used were as follows: anti‐phospho‐AMPKα (diluted 1:1000; Thr172, 40H9, #2535), anti‐AMPKα (diluted 1:1000; D5A2, #5831), anti‐phospho‐Acetyl‐CoA Carboxylase (diluted 1:1000; Ser79, D7D11, #11818), anti‐GAPDH (diluted 1:5000; D16H11, #5174), anti‐β‐tubulin (diluted 1:5000; #2146), anti‐β‐actin (diluted 1:5000; 8H10D10, #3700) (all from Cell Signaling Technology), anti‐acetyl‐CoA Carboxylase (diluted 1:1000; clone 7D2.2; Merck KGaA, Darmstadt, Germany), clone KM2076 anti‐Klotho (diluted 1:500; #SCE‐KO603; Hölzel Diagnostika, Cologne, Germany), goat anti‐rabbit IgG HRP‐linked (diluted 1:5000; Cell Signaling Technology), goat anti‐mouse IgG HRP‐linked (diluted 1:5000; Abcam, Cambridge, UK), and goat anti‐rat IgG HRP‐linked antibody (diluted 1:5000; Novus Biologicals, Wiesbaden, Germany). Finally, proteins were visualized using Clarity Western (Bio‐Rad Laboratories) or Westar Hypernova (Cyanagen, Bologna, Italy) ECL substrate. Bands were detected by ChemiDoc MP Imaging System (Bio‐Rad Laboratories) and signal intensities were quantified using Image Lab Software (Version 6.1, Bio‐Rad Laboratories). Data are shown as ratios of target protein over loading control [glyceraldehyde‐3‐phosphate dehydrogenase (GAPDH), β‐actin or β‐tubulin] or phosphorylated protein over total protein, normalized to control.

### Statistics

Data are shown as arithmetic means ± standard error of the mean (SEM), with *n* indicating the number of independent cell culture experiments. All data were tested for normality (Shapiro–Wilk test). Statistical comparisons of two groups were made by two‐tailed paired Student's *t*‐test. For more than two groups, nonparametric Friedman test followed by Dunn's multiple comparison test or two‐way ANOVA followed by Tukey's multiple comparison test were applied as indicated in the figure legends. Differences with *P* < 0.05 were considered statistically significant. GraphPad Prism 10 (Version 10.2.2; GraphPad Software Inc., San Diego, CA, USA) was used for statistical analyses.

## Results

**Fig. 1:**
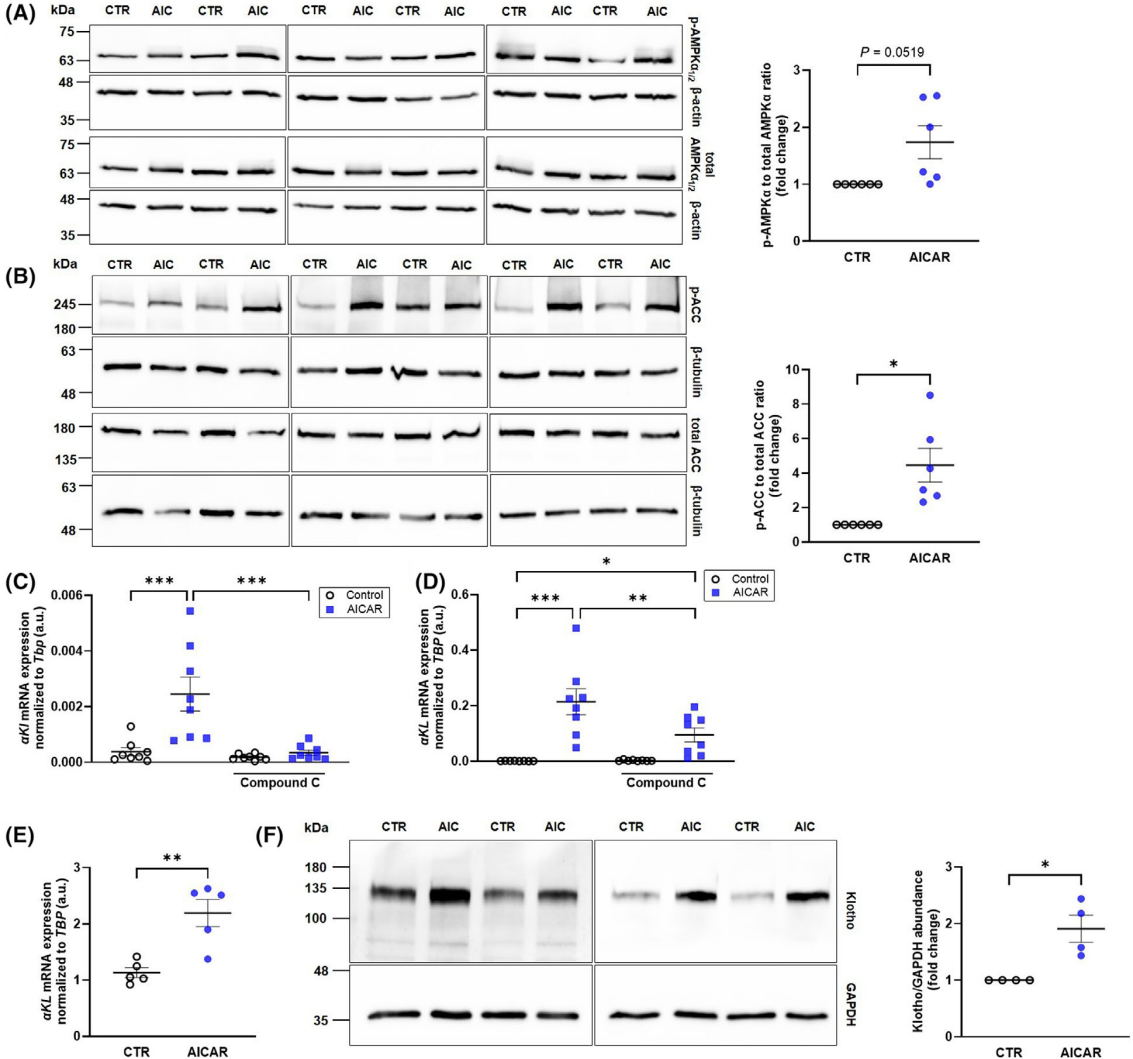
AMP‐dependent kinase (AMPK) activation induces αKlotho gene expression in normal rat kidney 52E (NRK‐52E) and Madin–Darby canine kidney (MDCK) cells and αKlotho protein expression in human kidney 2 (HK‐2) cells. (A) Original western blots (left panel) and densitometric analysis (right panel; *n* = 6) of phospho‐AMPKα_1/2_, total AMPKα_1/2_, and loading control β‐actin protein abundance in NRK‐52E cells treated with or without 1 mm 5‐aminoimidazole‐4‐carboxamide ribonucleoside (AICAR) for 6 h. (B) Original western blots (left panel) and densitometric analysis (right panel; *n* = 6) of phospho‐ACC, total acetyl‐CoA carboxylase (ACC), and loading control β‐tubulin protein abundance in NRK‐52E cells treated with or without 1 mm AICAR for 48 h. Arithmetic means ± SEM of αKlotho mRNA expression relative to Tbp in NRK‐52E cells (C, *n* = 8) or MDCK cells (D, *n* = 8) incubated with (blue squares) or without (white circles) AMPK activator AICAR (1 mm) in the presence or absence of AMPK inhibitor compound C for 48 h (1 μm, C) or 24 h (20 μm, D). Arithmetic means ± SEM (E, *n* = 5) of αKlotho mRNA expression relative to TBP in HK‐2 cells incubated with or without (white circles) 1 mm AICAR for 48 h. (F) Original western blots (left panel) and densitometric analysis (right panel; *n* = 4) of αKlotho and loading control GAPDH in HK‐2 cells treated with or without 1 mm AICAR for 38 h. *P < 0.05, **P < 0.01, ***P < 0.001. (A, B, E, F: Paired *t*‐test; C, D: two‐way ANOVA). a.u., arbitrary units; AIC, AICAR; GAPDH, glyceraldehyde‐3‐phosphate dehydrogenase.

At first, we carried out experiments using NRK‐52E cells to investigate αKlotho expression. We treated the cells for 6 h with 5‐aminoimidazole‐4‐carboxamide ribonucleoside (AICAR), an agent mimicking the stimulating effect of AMP on AMPK, and determined phospho‐AMPKα (as a measure of AMPK activity) and total AMPKα_1/2_ protein levels by western blotting. As depicted in Fig. 1A, treatment with AICAR enhanced phospho‐AMPKα protein abundance, an effect almost reaching statistical significance. Also downstream effector acetyl‐CoA carboxylase (ACC) became phosphorylated following AICAR treatment (Fig. 1B). Next, we quantified αKlotho transcripts by qRT‐PCR. Treatment with AICAR resulted in a pronounced and significant increase in αKlotho mRNA abundance (Fig. 1C). In order to test whether the AICAR effect was indeed dependent on AMPK, we applied AMPK inhibitor compound C. As illustrated in Fig. 1C, co‐incubation with compound C significantly attenuated the AICAR effect on αKlotho transcripts. Hence, enhanced AMPK activity resulted in upregulation of αKlotho gene expression in NRK‐52E cells. Next, we studied the impact of AMPK on αKlotho expression in another cell model, MDCK cells, a distal tubular cell line from canine kidney. Also in MDCK cells, treatment with AICAR led to significant enhancement of αKlotho mRNA abundance (Fig. 1D). Similar to NRK‐52E cells, this effect was blunted by AMPK inhibitor compound C (Fig. 1D). Another series of experiments was performed in human kidney 2 (HK‐2) cells. Treatment with AICAR also upregulated αKlotho mRNA expression in this cell line (Fig. 1E). Importantly, this effect was paralleled by a surge of αKlotho protein expression as determined by western blotting (Fig. 1F).

**Fig. 2:**
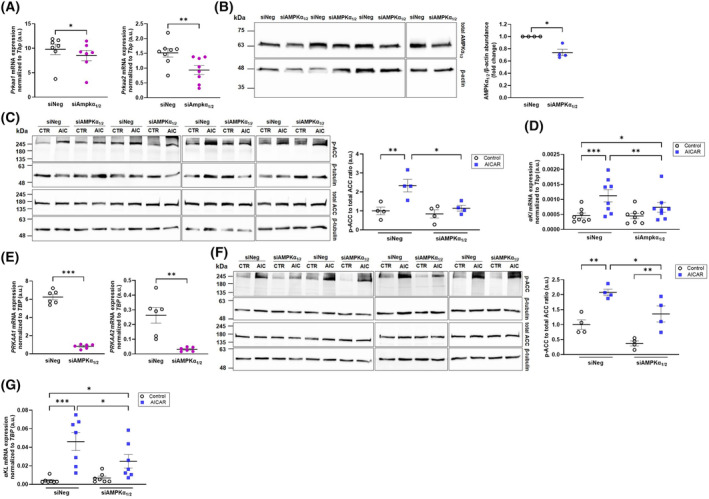
Effect of 5‐aminoimidazole‐4‐carboxamide ribonucleoside (AICAR) on αKlotho mRNA expression in normal rat kidney 52E (NRK‐52E) and Madin–Darby canine kidney (MDCK) cells is blunted by AMPKα_1/2_ silencing. Arithmetic means ± SEM of Ampkα_1_ (A, E; left panels), Ampkα_2_ (A, E; right panels), and αKlotho (D, G) mRNA expression relative to Tbp in NRK‐52E cells (A, *n* = 7–8; D, *n* = 8) and MDCK cells (E, *n* = 6; G, *n* = 7) transfected for 24 h with either nontargeting siRNA (siNeg) or siRNA specifically targeting Ampkα_1/2_ (siAmpkα_1/2_) and treated further for 48 h (D) or 24 h (G) with (blue squares) or without (white circles) AMP‐dependent kinase (AMPK) activator AICAR (1 mm). (B) Original western blots (left panel) and densitometric analysis (right panel; *n* = 4) of total AMPKα_1/2_ and loading control β‐actin protein abundance in NRK‐52E cells treated for 72 h with 50 nm siNeg or siAmpkα_1/2_. Original western blots (C, F; left panel) and densitometric analysis (right panel; *n* = 4) of phospho‐ACC, total acetyl‐CoA carboxylase (ACC) and loading control β‐tubulin protein abundance in NRK‐52E cells treated with 50 nm (C) or MDCK cells treated with 25 nm siNeg or siAmpkα_1/2_ (F) in the presence or absence of 1 mm AICAR (24–48 h). *P < 0.05, **P < 0.01, ***P < 0.001. (A, B, E: Paired *t*‐test; C, D, F, G: two‐way ANOVA). a.u., arbitrary units; AIC, AICAR.

The next series of experiments utilized siRNA‐mediated knockdown of AMPKα_1_ and AMPKα_2_ gene expression. Silencing was efficient (Fig. 2A) in NRK‐52E cells as demonstrated by downregulation of AMPKα_1_ (encoded by *Prkaa1*; Fig. 2A, left panel) and AMPKα_2_ (encoded by *Prkaa2*; Fig. 2A, right panel) subunit. Silencing also significantly lowered AMPKα_1/2_ protein abundance (Fig. 2B). In NRK‐52E cells exposed to siRNA targeting AMPKα_1/2_, the AICAR effect on phospho‐ACC was blunted compared with cells treated with nonsense siRNA (Fig. 2C), suggesting that silencing of AMPKα_1/2_ was functionally relevant. In cells exposed to nonsense siRNA, AICAR readily upregulated αKlotho gene expression (Fig. 2D). Treatment with specific siRNA targeting AMPKα_1_ and AMPKα_2_, however, led to a significantly smaller AICAR‐dependent upregulation of αKlotho transcripts compared with NRK‐52E cells treated with nonspecific siRNA (Fig. 2D). Similar effects could be observed in MDCK cells: Silencing was again efficient (Fig. 2E). Moreover, similar to NRK‐52E cells, the AICAR effect on phospho‐ACC was significantly attenuated in MDCK cells exposed to siRNA targeting AMPKα_1/2_, compared with cells treated with nonsense siRNA (Fig. 2F). Also, AICAR treatment increased αKlotho mRNA levels in cells incubated with nonsense siRNA (Fig. 2G). Preincubation with siRNA specifically targeting AMPKα_1/2_ led to an AICAR effect which was again significantly reduced compared with MDCK cells exposed to negative siRNA (Fig. 2G).

**Fig. 3:**
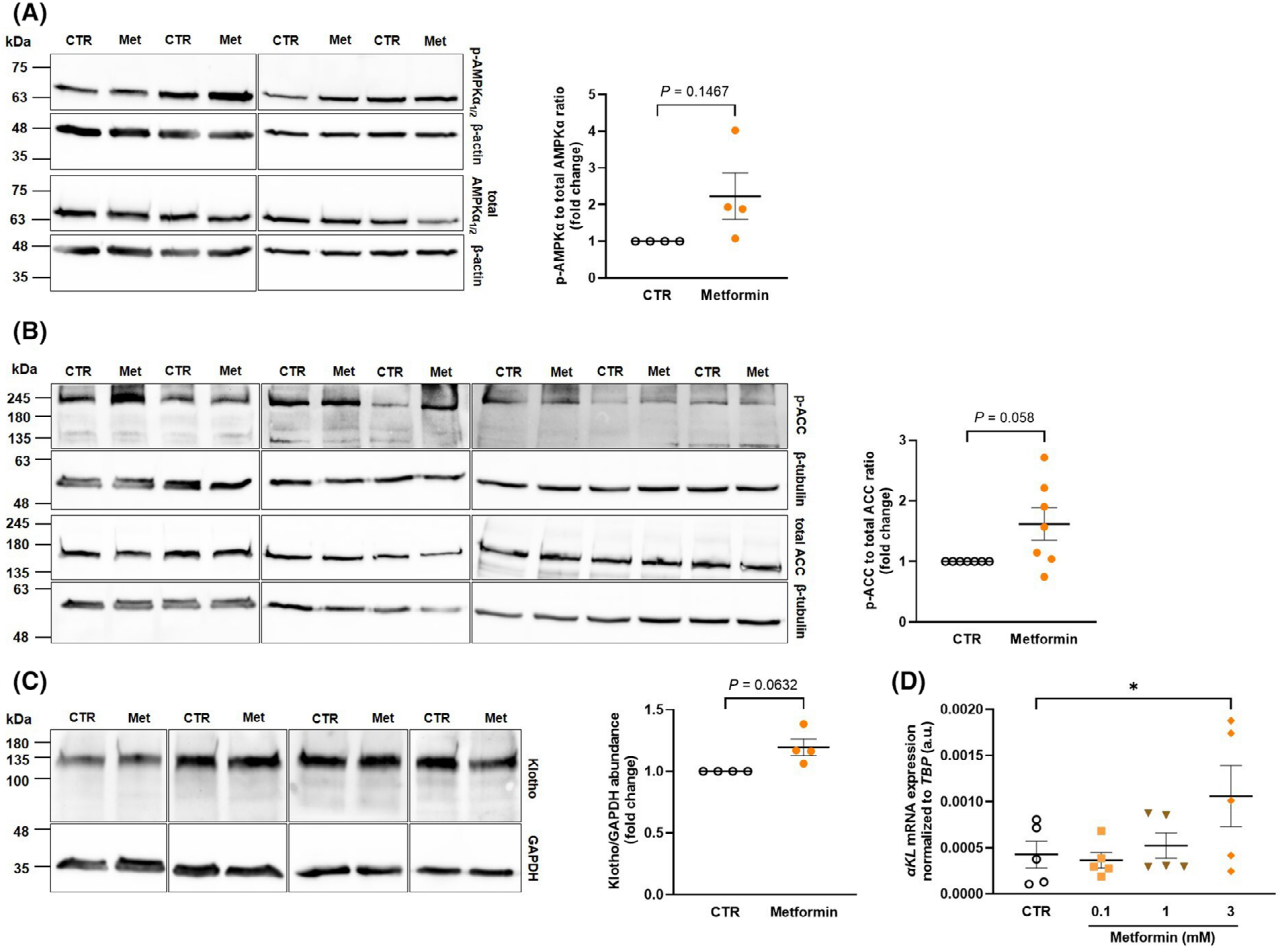
Biguanide metformin activates AMPKα in human kidney 2 (HK‐2) cells and induces αKlotho transcription and protein expression in HK‐2 and Madin–Darby canine kidney (MDCK) cells. (A) Original western blots (left panel) and densitometric analysis (right panel; *n* = 4) of phospho‐AMPKα_1/2_ and total AMPKα_1/2_ protein over loading control β‐actin expression in HK‐2 cells treated with or without 3 mm metformin hydrochloride for 24 h. (B) Original western blots (left panel) and densitometric analysis (right panel; *n* = 7) of phospho‐ACC, total acetyl‐CoA carboxylase (ACC) and loading control β‐tubulin protein abundance in HK‐2 cells treated with or without 3 mm metformin hydrochloride for 24 h. (C) Original western blots (left panel) and densitometric analysis (right panel; *n* = 4) of αKlotho and loading control GAPDH in HK‐2 cells treated with or without 3 mm metformin hydrochloride for 24 h. (D) Arithmetic means ± SEM (*n* = 5) of αKlotho mRNA expression relative to TBP in MDCK cells incubated with or without (white circles) metformin hydrochloride at the indicated concentrations for 24 h. *P < 0.05. (A, B, C: Paired *t*‐test; D: Friedman test). a.u., arbitrary units; GAPDH, glyceraldehyde‐3‐phosphate dehydrogenase; Met, metformin hydrochloride.

**Fig. 4:**
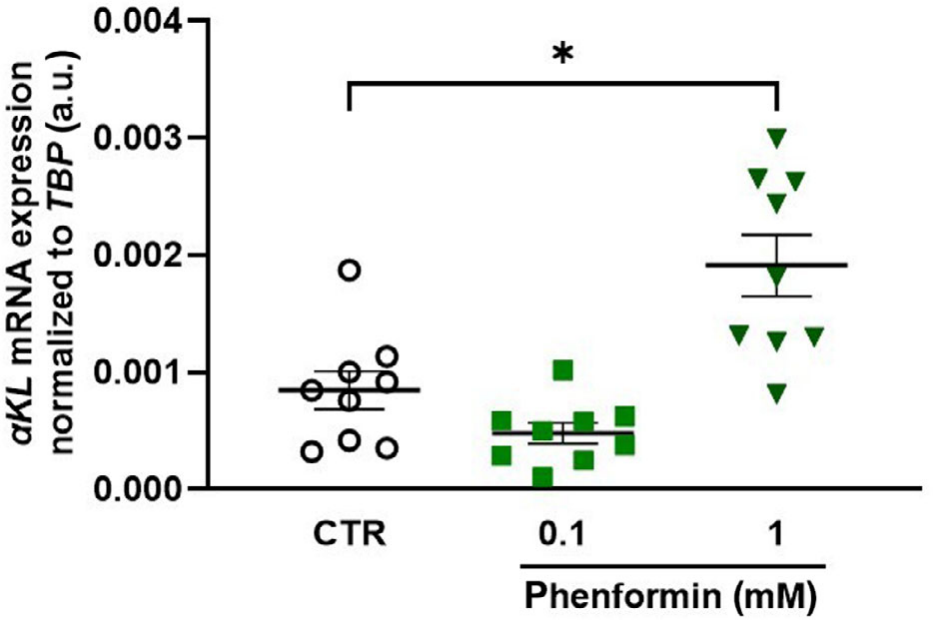
Biguanide phenformin induces αKlotho gene expression in Madin–Darby canine kidney (MDCK) cells. Arithmetic means ± SEM (*n* = 9) of αKlotho mRNA expression relative to TBP in MDCK cells incubated with or without (white circles) phenformin hydrochloride at the indicated concentrations for 24 h. *P < 0.05. (Friedman test). a.u., arbitrary units.

Biguanide metformin is a further AMPK activator that is broadly used in the treatment of type II diabetes [34]. Studying HK‐2 cells, we explored whether also metformin impacts on αKlotho. First, we demonstrated that metformin tends to upregulate phospho‐AMPKα_1/2_ expression reflecting AMPK activity, an effect, however, missing statistical significance (Fig. 3A). Metformin also tended to upregulate phospho‐ACC, an effect almost reaching significance (Fig. 3B). Importantly, also metformin upregulated αKlotho protein expression (Fig. 3C). Moreover, as demonstrated in Fig. 3D, a 24 h‐treatment with metformin upregulated the abundance of αKlotho mRNA in MDCK cells. Phenformin, another biguanide, similarly enhanced αKlotho gene expression in MDCK cells within 24 h (Fig. 4).

**Fig. 5:**
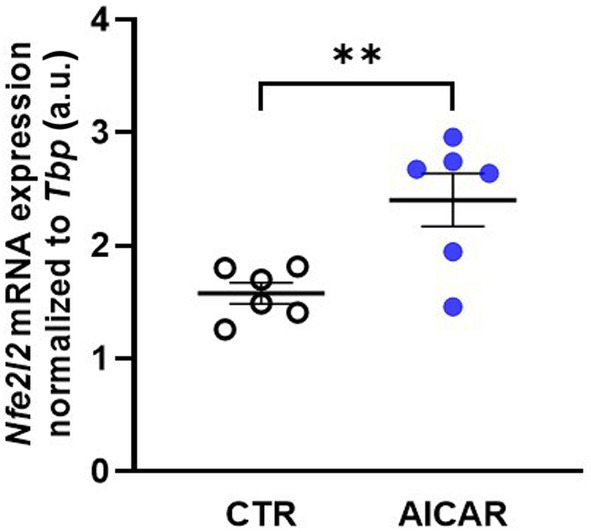
5‐aminoimidazole‐4‐carboxamide ribonucleoside (AICAR) treatment induces Nfe2l2 gene expression in normal rat kidney 52E (NRK‐52E) cells. Arithmetic means ± SEM (*n* = 6) of Nfe2l2 mRNA expression relative to Tbp in NRK‐52E cells incubated with or without (white circles) 1 mm AICAR for 48 h. **P < 0.01. (Paired *t*‐test). a.u., arbitrary units.

Some of the beneficial effects of αKlotho are mediated by upregulation of transcription factor Nrf2 (encoded by *Nfe2l2*) [35]. In order to study whether the AICAR effect on αKlotho is functionally relevant, we measured *Nfe2l2* transcripts in NRK‐52E cells. As shown in Fig. 5, AICAR upregulated *Nfe2l2*, an effect in line with αKlotho enhancing *Nfe2l2* expression [35].

## Discussion

Using NRK‐52E, MDCK, and HK‐2 cells, our experiments revealed pharmacological activation of AMPK as a powerful stimulator of antiaging αKlotho gene expression. Upregulation of AMPK activity with three different agonists, AICAR, metformin, or phenformin was similarly capable of enhancing αKlotho gene expression. Moreover, silencing of AMPKα_1/2_ or AMPK inhibition with compound C significantly blunted the AICAR effect on αKlotho. In HK‐2 cells, we also confirmed αKlotho protein upregulation following treatment with AICAR or metformin.

Importantly, AICAR indeed induced AMPK activity as demonstrated by enhancement of phospho‐AMPKα and phospho‐ACC protein abundance.

Pharmacological induction of αKlotho formation can be expected to have several beneficial effects: Higher αKlotho availability expands life span of mice by about 30% [16] and has been proven beneficial in several diseases: Acute kidney injury (AKI) and chronic kidney disease (CKD) are characterized by αKlotho deficiency [36], and αKlotho prevents progression from AKI to CKD [37] and protects from uremic cardiomyopathy [38]. Mechanistically, αKlotho is beneficial due to anti‐inflammatory [39], antioxidant [35], antifibrotic [40], and anticalcification [41] effects. Also in the cardiovascular system, higher αKlotho availability is desirable as it helps maintain vasculature health by improving endothelial function [42] and integrity [43]. αKlotho protects from uremic cardiomyopathy [44] and ischemia/reperfusion injury of the heart [45]. Therefore, therapeutic increase in αKlotho expression is a novel approach in cardiovascular disease [46]. αKlotho is neuroprotective [47] and a tumor suppressor [48]. Taken together, a feasible therapeutic intervention to enhance αKlotho expression as uncovered by our study is likely to have broad clinical implication.

In our study, AMPK activator AICAR turned out to be a particularly powerful enhancer of αKlotho gene expression. Independently of this novel effect, but in perfect line with it, health‐promoting effects of AICAR are well known and include improvements of insulin resistance [49], as well as positive renal [50], or cardiac effects [51]. In general, AMPK activity confers cardiac [52] or renal benefits [53] as does AMPK activator metformin [54, 55, 56, 57]. It is striking that αKlotho, AMPK activation, or AMPK activator metformin exert beneficial effects in the same organs. Therefore, our main finding, that is, stimulation of αKlotho through AMPK, may therefore shed new light on the mechanisms underlying AMPK‐dependent or metformin‐dependent health benefits. However, it must be kept in mind that metformin is not a specific AMPK activator and may have further AMPK‐independent effects [58]. Therefore, metformin‐dependent upregulation of αKlotho may also be, at least in part, due to further, AMPK‐independent pathways.

Whereas our major finding, that is, upregulation of αKlotho by AMPK is novel, it has been described by others before that αKlotho induces AMPK signaling [59, 60, 61], suggesting that at least some of the beneficial effects of αKlotho may be mediated by AMPK. Our study may suggest mutual regulation of AMPK and αKlotho, two molecules known for their health beneficial effects.

It is a limitation of our study that it is solely based on cell culture experiments. Moreover, different loading controls were used for the various blots of this study, which has to be taken into account when interpreting the results. Clearly, further *in vivo* studies are needed to define the exact role of AMPK‐dependent regulation of αKlotho. We demonstrated that AICAR increased expression of *Nfe2l2* encoding Nrf2, a transcription factor mediating some beneficial effects of αKlotho [35]. Therefore, this effect can, at least in part, be attributed to the AMPK effect on αKlotho. However, further functional studies are warranted.

Taken together, our study shows that AMPK activation is a powerful stimulator of antiaging αKlotho gene expression. The results are likely to contribute to beneficial effects associated with pharmacological AMPK activation and/or treatment with metformin.

## Conflict of interest

MFö received honoraria from Kyowa Kirin without relevance for this study. The other authors have no conflict of interest to declare.

## Author contributions

MFö and MFe designed the research. MFö, MFe, JV, LH, and LW interpreted data. MFö, JV, and LW wrote the manuscript. JV and LW performed the research and analyses. All authors read and approved the final manuscript.

## Data Availability

The data that support the findings of this study are available from the corresponding author [michael.foeller@uni-hohenheim.de] upon reasonable request.
